# Surgical Management of Pediatric Gastroesophageal Reflux Disease

**DOI:** 10.1155/2013/863527

**Published:** 2013-05-09

**Authors:** Hope T. Jackson, Timothy D. Kane

**Affiliations:** ^1^Department of Surgery, The George Washington University School of Medicine & Health Sciences, Washington, DC, USA; ^2^Division of Pediatric Surgery, Department of Surgery, Sheikh Zayed Institute for Pediatric Surgical Innovation, Children's National Medical Center, Washington, DC, USA; ^3^Surgical Residency Training Program, Children's National Medical Center, Division of Pediatric Surgery, 111 Michigan Avenue, NW Washington, DC 20010-2970, USA

## Abstract

Gastroesophageal reflux (GER) is common in the pediatric population. Most cases represent physiologic GER and as the lower esophageal sphincter (LES) matures and a solid diet is introduced, many of these patients (>65%) experience spontaneous resolution of symptoms by two years of age. Those who continue to have symptoms and develop complications such as failure to thrive, secondary respiratory disease, and others are classified as having gastroesophageal reflux disease (GERD). Goals of GERD treatment include the resolution of symptoms and prevention of complications. Treatment options to achieve these goals include dietary or behavioral modifications, pharmacologic intervention, and surgical therapy. This paper will review the clinical presentation of GERD and discuss options for surgical management and outcomes in these patients.

## 1. Introduction

Gastroesophageal reflux (GER) is a common and often benign occurrence in the pediatric population that refers to the regurgitation of gastric contents into the esophagus. The majority of these patients (>65%) will experience spontaneous resolution of their symptoms by two years of age [1–3]. Those who continue to have symptoms and develop complications such as failure to thrive, secondary respiratory disease, laryngospasm, esophagitis, and esophageal strictures are classified as having gastroesophageal reflux disease (GERD). The overall goals for the treatment of GERD are to relieve symptoms, maintain remission of symptoms, and manage or prevent complications. Treatment options to achieve these goals include dietary or behavioral modifications, pharmacologic intervention, and surgical therapy. Increased understanding of GERD pathophysiology has led to improved diagnostic techniques, pharmacologic agents, and invariable approaches to surgical management [4]. This paper will review the classification of physiologic and pathologic GER and clinical presentation and diagnosis of GERD as well as discuss options for surgical management and outcomes in these patients. 

## 2. Classification

### 2.1. Physiologic GER and Pathologic GER/GERD

Up to 60% of healthy infants 0–6 months of age experience occasional refluxing of gastric contents into the esophagus. This percentage declines to 5% at one year of age [5]. The mechanism of reflux is believed to be due to an immature lower esophageal sphincter (LES) and a predominately liquid diet and it is considered physiologic. As the LES matures and solids are introduced into the diet, reflux resolves, typically by 12 months of age [6]. Those children who do not experience resolution of their reflux may go on to develop GERD which describes the complications that can result from persistent GER (i.e., secondary respiratory disease, apnea, acute-life threatening events (ALTE), and esophageal stricture). The pathophysiology of GERD is believed to have anatomical (short esophagus, stricture, and hiatal hernia) and/or functional components (pharmacologic agents, poor dietary habits, and abnormal gastric motility) [7].

## 3. Clinical Presentation/Diagnosis

The clinical symptoms of reflux that lead to GERD may vary according to the age of the child. Infants commonly present with regurgitation, vomiting, and irritability while the older child or adolescent may more commonly present with dysphagia, epigastric/substernal pain, and heartburn [17]; see Table 1. Management of GERD in both adults and children is based on disease severity, the degree of symptoms, and presence or absence of complications of GER determined by diagnostic evaluation [4]. Given these variables, it is no surprise that GERD is rarely diagnosed solely on the basis of one diagnostic test, but usually a combination of studies. While GERD can be diagnosed by typical history and physical examination findings as a basis for a trial of therapy, typical symptoms are not always present and do not always predict which patients will respond to treatment. See Table 2 for the most commonly used diagnostic tests in the evaluation of GERD.

## 4. Management

The management of children with GERD initially begins with nonoperative measures that often result in resolution of symptoms. The goals of medical management include lifestyle modifications, acid-suppressive medications designed to alter gastric pH, and prokinetic agents that seek to improve the transit of gastric contents. Lifestyle modifications consist of formula changes and thickened feeds in infants and reduced caffeine intake and weight reduction in adolescents. Adolescents also make up a portion of the smoking population. While pediatricians should counsel against smoking and advocate for cessation regardless of the presence of GERD, it is even more important in those patients experiencing GERD symptoms.

Surgical management of GERD typically becomes necessary in presence of GER complications and/or failed medical therapy. It is considered for the patient with esophagitis, stricture, pulmonary symptoms such as asthma and recurrent pneumonia, and finally those with failure or inadequate response to medical therapy associated with neurologic handicaps [18, 19]. See Table 3 for a list of common indications for surgical management. Antireflux procedures are usually performed to eradicate the reflux of gastric contents into the esophagus which should control GERD related symptoms, prevent complications, and permit adequate caloric intake to achieve growth [20]. This is achieved by reestablishing the gastroesophageal barrier through creation of a partial or complete valve mechanism at the gastroesophageal junction (fundoplication) [7]. Over the years, laparoscopic antireflux procedures (first reported in children in 1993) have replaced the open approach to become the primary surgical approach for the treatment of GERD [11, 21].

### 4.1. Fundoplication

**Figure 1:**
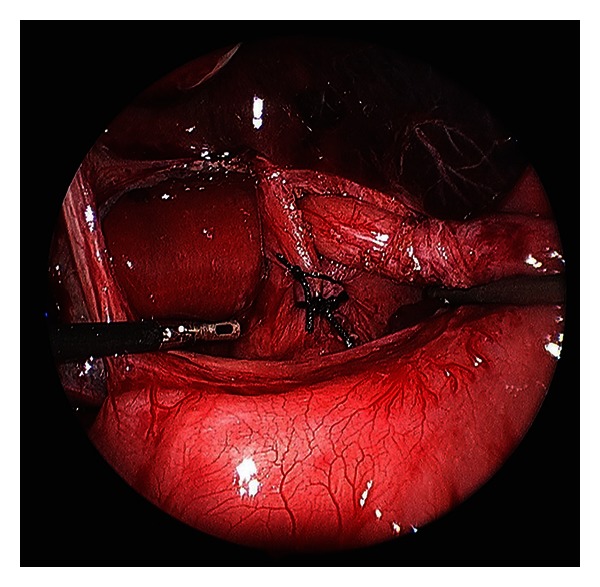
Laparoscopic Nissen fundoplication technique. Esophageal mobilization with creation of a retroesophageal window and crural approximation (sutures).

**Figure 2:**
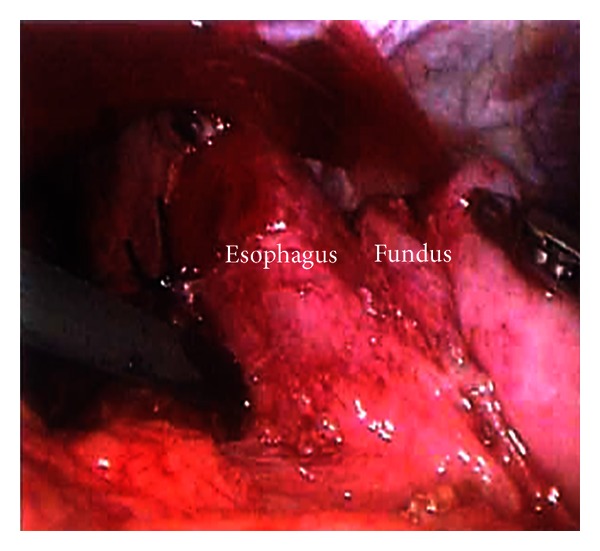
Laparoscopic Nissen fundoplication technique. Creation of the fundoplication “wrap” by passing the fundus of the stomach behind the esophagus (through the retroesophageal window). This is called the “shoe shine maneuver.”

**Figure 3:**
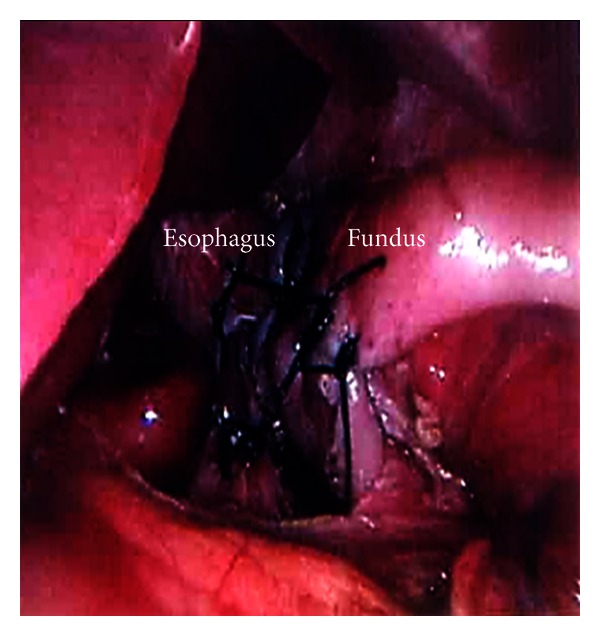
Laparoscopic Nissen fundoplication technique. Completed 360° fundoplication.

Fundoplication provides definitive treatment for GERD and is highly effective in most circumstances. The fundus of the stomach can be wrapped around the distal esophagus either 360 degrees (i.e., Nissen fundoplication) or to lesser degrees (i.e., Thal or Toupet fundoplication). Initially described in 1954 by Rudolph Nissen, the Nissen fundoplication has evolved to become the standard operation for the surgical treatment of GERD in children and adults [17]. Nissen described the procedure as a 360 degree gastric fundoplication around the distal esophagus for a distance of 4-5 centimeters. This provided solid control of reflux but was associated with numerous side effects that encouraged modifications to the procedure. These changes included using only the gastric fundus to surround the esophagus and limiting the length of the fundoplication to 1 to 2 centimeters [22]. The essential steps to performing both an open and laparoscopic fundoplication can be found in Table 4. Figures 1, 2, and 3 also highlight key steps of the laparoscopic fundoplication. 

### 4.2. Partial Fundoplication

Partial fundoplication procedures involve wrapping the distal esophagus to a lesser degree than required in the Nissen procedure (e.g., 270 degrees). Partial wraps are often performed in those patients with esophageal motility disorders to prevent dysphagia that may result from a complete fundoplication. The most commonly performed partial fundoplications are Toupet (posterior) and Thal (anterior). The steps of the Toupet procedure are similar to the Nissen; however, once the fundus is mobilized posteriorly around the esophagus, the edges of the fundus are sutured to the right and left sides of the distal esophagus which ensures that the wrap only partially encircles the esophagus (posteriorly). A Thal is performed by approximating the hiatus posterior to the esophagus and then the fundus is sutured anteriorly with fixation to the esophagus and the diaphragm (anteriorly). 

### 4.3. Nissen versus Partial Fundoplication

Currently, there are four large retrospective studies in the literature that compare the different laparoscopic fundoplication techniques in children. In 2001, Esposito et al. [23] showed that laparoscopic fundoplication was feasible even in pediatric patients less than one year of age. In 2006 Esposito et al. then observed no statistical significance in outcome between laparoscopic Nissen, Toupet, and Thal procedures in neurologically normal children in the hands of experienced pediatric surgeons [24] (Table 5). Similarly, Chung and Georgeson [12] and Steyaert et al. [13] reported that Nissen and Toupet procedures were comparable with regard to reflux control. Among the four studies, the reoperation rate ranged between 2.1% and 11.1%, with the highest incidence reported by Esposito et al. [23] in 36 infants [12, 13, 23, 24]. Kubiak et al. [15] published the first prospective randomized trial seeking to compare the long-term outcomes and control of symptoms after Nissen and Thal fundoplications in children. In this study, the Nissen fundoplication had a significantly lower recurrence rate than the Thal (5.9% versus 15.9%) in patients with underlying neurological disorders. There was no significant difference between the fundoplications in normal children. In terms of control of symptoms, the incidence of postoperative dysphagia was similar in both groups, but significantly more patients in the Nissen group required intervention for severe dysphagia (11.8% versus 2.4%). In those patients who had a recurrence of moderate symptoms, there was no significant difference in the need to restart antireflux medication between both groups. 

### 4.4. Learning Curves for Laparoscopic Fundoplication

 As with any surgical technique, a period of learning is expected to master approach, technique, and avoid complications. With respect to antireflux surgery (complete and partial fundoplication), the laparoscopic approach requires the need for intracorporeal suturing and specific dissection and mobilization techniques that can be challenging to even the most experienced surgeon.

 There are both adult and pediatric studies that address the learning curves associated with laparoscopic surgery. Watson et al. [25] reported an institutional learning curve of 50 procedures and individual learning curves of 20 operations from an initial experience of 280 laparoscopic antireflux procedures in adults. They also noted that the adverse effects of the learning curve could be avoided if new surgeons performed their initial cases under the direct supervision of an experienced surgeon.

 In children, Meehan and Georgeson [26] looked at the learning curve in their first 160 cases of laparoscopic fundoplications and suggested a learning curve in terms of conversion to open and operative times between 20 and 25 cases. In his series of 220 procedures, Rothenberg [27] also reported an estimated learning curve for laparoscopic Nissen fundoplication to be between 20 and 50 cases. 

 As this learning period is to be expected, the presence/consultation of a senior surgeon during this period may mitigate longer operative times and increased risk of surgical complications. It is also important to note that the surgeon's learning curve extends as technique improves and more complicated patients are referred for operation [28]. 

### 4.5. Fundoplication Plus Gastrostomy

Though a large number of patients who require a fundoplication also receive a gastrostomy, children with intact swallowing or those who were not dependent on gastrostomy or tube feeding before antireflux surgery are candidates for fundoplication alone [7]. In those children with a preexisting gastrostomy, the tube can interfere with dissection of the hiatus or performance of the wrap and create too much tension on the fundus to perform an adequate fundoplication. Therefore, if leaving the old gastrostomy tube in place will compromise the performance of the fundoplication, the authors prefer to take down the old site, close with suture repair, and replace it at the end of the procedure. 

### 4.6. Fundoplication Plus Pyloroplasty

Delayed gastric emptying is associated with a significant number of patients with GERD and has also been reported in the postoperative period [7]. This has brought into question whether a pyloroplasty should be performed at the time of fundoplication. The outcomes of children who have undergone Nissen fundoplication with pyloroplasty are similar to those who have been treated without pyloroplasty in terms of recurrence of symptoms, reoperation, and readmission [29]. However, short-term postoperative complications have been reported to be higher when pyloroplasty was added to the antireflux procedure [29]. Lastly, improved gastric emptying after fundoplication as documented by preoperative and postoperative gastric emptying scans in both adults and children has led to the common practice for surgeons to perform fundoplication without pyloroplasty [30, 31].

### 4.7. Gastrostomy

The challenges that result from failed fundoplication have led to the implementation of alternative surgical management strategies for GERD [17]. Many children who require gastrostomy placement often have coexistent GER [32]. This is particularly true in neurologically impaired children. In the past, those requiring a gastrostomy tube would also receive an antireflux procedure at the time of tube placement. Neurologically impaired children have been shown to have a poorer prognosis following antireflux surgery compared to neurologically normal children [33]. Consequently, several studies sought to challenge the notion that an antireflux surgery should still be performed irrespective of GER symptom resolution with gastrostomy tube placement. A retrospective analysis by Wilson et al. [32] in 2006 reported that symptoms of GERD were alleviated in 68% of children with gastrostomy alone. Fourteen percent of those who had persistent GER symptoms responded with the addition of antireflux medications and only 7% of the included patients eventually required an antireflux procedure. While the mechanism for symptom improvement is unclear, this study does suggest that it may be a viable surgical alternative, particularly in neurologically impaired children that may have other coexisting medical conditions that increase preoperative surgical risk. This issue is still controversial, however, as a retrospective analysis by Srivastava et al. in 2009 revealed that reflux-related hospital admissions for neurologically impaired children who underwent Nissen fundoplication were reduced compared to hospital admissions before-fundoplication [34].

### 4.8. Gastrojejunal Feeding

Of all the surgical management procedures, the least invasive is the placement of a nasojejunal or gastrojejunal feeding tube. This allows the stomach to be bypassed, preventing food contents from entering the esophagus, and often results in symptom improvement. This technique is limited, however, as a long-term management strategy. Patient comfort, tube dislodgement, inability to bolus feed, the need for lifelong antireflux medications, and rarely enteroenteric intussusceptions are often cited as disadvantages to this management option. Some literature suggests that this option is best reserved for those neurologically impaired children with increased operative risk [35].

### 4.9. Total Esophagogastric Dissociation

Originally described by Bianchi in 1997, total esophagogastric dissociation emerged as a surgical option for those who have repeatedly failed attempts at fundoplication or have severe neurologic impairment [36]. This procedure permanently eliminates GERD by transecting the esophagus from the stomach and creating an esophagojejunal (EJ) anastomosis. The biliopancreatic limb is then anastomosed to the jejunal loop approximately 30 cm distal to the EJ anastomosis in order to drain the gastric contents. This procedure was recently shown to be feasible laparoscopically in children [37]. In addition, gastric feedings may still be utilized via a gastrostomy tube in the remnant stomach without the risk of reflux.

### 4.10. Endoscopic Approaches

During the past few years, a number of endoscopic procedures aimed at improvement of the barrier function of the lower esophageal sphincter (LES) have emerged. In general, these endoscopic techniques use two different approaches to reduce reflux and improve the gastroesophageal barrier function. In one approach the GE junction can be tightened by the endoscopic creation of plications and in another radiofrequency energy is delivered to the lower esophagus and cardia to obtain collagen remodeling and augment LES pressure. 

Endoluminal gastroplication involves the endoscopic creation of multiple folds or plicae in the stomach below the LES. In 2004, Thomson et al. [38] reported their initial experience performing this procedure in children. In 2008, they reported their medium-term outcome which showed 88% of patients symptom free with no need for antireflux medications at 1 year, 56% at 3 years and a rate of symptom recurrence requiring reoperation of 25% at 3 years [39]. There are no data regarding the long-term outcomes of gastroplication in children. 

The next endoscopic procedure that has been described in children is the Stretta procedure. In this procedure, radiofrequency energy is delivered in multiple levels around the GE junction (approximately 2-3 cm). The intent is to create a high pressure zone that reduces reflux through scarring of the lower esophagus. This scarring not only creates a high pressure zone but it also causes a decrease in the number of transient LES relaxations due to disruption of adjacent vagal afferent fibers [40]. Studies in adults show questionable improvement in GERD symptoms, patient satisfaction, quality of life, and need for medication sustained over 4 years of followup, and the use of Stretta in children is based on type III evidence [9, 17, 41]. At this point based on the limited data and lack of long-term outcomes, both the Stretta and endoluminal gastroplication techniques are included for historical perspective and context. They cannot be recommended as surgical options for the treatment of GERD in children.

## 5. Surgical Complications

Antireflux surgery complications can be divided into short and long-term events. Short-term will describe intraoperative and initial postoperative period complications. Long-term complications will refer to those complications developing several months to years after the initial procedure.

### 5.1. Intraoperative Complications

Bleeding, esophageal and gastric perforation (all repaired laparoscopically), vagus nerve injury, bowel injury, and pneumothorax have all been reported as intraoperative complications of laparoscopic antireflux surgery. The reported rate of these complications is between 0.5% and 11% [42–44].

### 5.2. Postoperative Complications

The challenge of any antireflux procedure is to reestablish the gastroesophageal barrier and eradicate symptoms of reflux without inducing dysphagia and hyperflatulence, symptoms that often characterize wraps that have been too tightly placed. Complications of surgery in the initial postoperative period are uncommon but include dysphagia and gas bloat. Dysphagia rates are reported to range from <1% to 23% [15, 42]. For dysphagia, the child is kept on liquid and semisolid foods until the dysphagia resolves which usually occurs by 3 weeks following the operation. As mentioned earlier in this paper, while dysphagia rates have been reported to be similar across all fundoplication types [24], the Nissen fundoplication has been shown to have a higher rate of severe dysphagia that required intervention than those patients who received a Thal fundoplication [15].

### 5.3. Long-Term Complications

**Figure 4:**
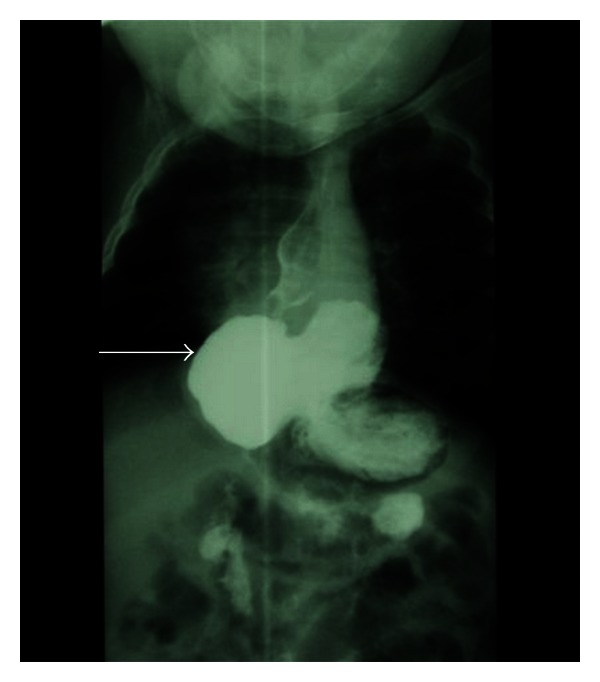
Upper gastrointestinal series. This imaging study reveals a slipped wrap with intrathoracic herniation (arrow).

Failed laparoscopic fundoplication defined as abnormal pH studies with symptoms has been shown to occur in 2% of neurologically normal and in up to 12% of neurologically impaired children [45]. Recurrence or persistence of reflux symptoms (i.e., heartburn and regurgitation) and postoperative persistent dysphagia are the most common indicators for failure of Nissen fundoplication. See Table 6 for common causes of fundoplication failure and Figure 4 for radiographic imaging which shows a slipped fundoplication with intrathoracic herniation. 

When patient symptoms persist, a “redo” fundoplication, whether open or laparoscopic, has been shown to be a safe option in the hands of an experienced pediatric surgeon with a 2-year failure rate of 6% [46]. While a redo fundoplication is feasible, it should be noted that successful repair can be technically challenging and entails extensive adhesiolysis, esophageal mobilization, crural repair, and wrap reformation [43].

## 6. Postoperative Care

The postoperative care of for an uncomplicated patient involves advancement of diet to liquids on the first postoperative day. This includes those patients with a gastrostomy. Once the patients have tolerated liquids, they can be advanced to a pureed diet which they are to remain on for at least 3 weeks. Outpatient care should include documentation of weight gain, food tolerance, and resolution of symptoms. Routine postoperative imaging such as an upper GI series is only indicated in the case of recurrence of symptoms or evidence of recurrent GERD.

## 7. Summary

Gastroesophageal reflux is a common occurrence in the pediatric population. The majority of cases represent physiologic GER and as the LES matures and a solid diet is introduced, many of these patients (>65%) experience spontaneous resolution of their symptoms by two years of age. Those who continue to have symptoms and develop complications such as failure to thrive, secondary respiratory disease and others are classified as having GERD. Goals of treatment include the resolution of symptoms and prevention of complications. Treatment options to achieve these goals include dietary or behavioral modifications, pharmacologic intervention, and surgical therapy. Overall, management of GERD in both adults and children is based on disease severity, the degree of symptoms, and presence or absence of complications of GER determined by diagnostic evaluation. The laparoscopic Nissen fundoplication is the standard operation for the surgical treatment of GERD. Partial fundoplications can also be performed, particularly in cases of underlying esophageal motility disorders, but it has been shown in some studies to have a higher recurrence rate than the Nissen fundoplication. Other techniques include gastrojejunal feeding, gastrostomy, and total esophagogastric dissociation and have promising early results in children. Uncomplicated postoperative care for fundoplications include early advancement of diet to liquids then pureed and outpatient documentation of resolution of symptoms. Complications of surgery include both short term (intraoperative, postop dysphagia, and hyperflatulence) and long term (failed fundoplication). The learning curve for antireflux surgery is approximated to be between 20 and 50 cases but continues to extend as the surgeon is referred more complicated cases. In the case of failed fundoplication, a “redo” procedure is safe and appropriate in the hands of an experienced surgeon.

## Figures and Tables

**Table 1:** Common symptoms of infant and adolescent patients presenting with GERD [8].

Infants	Older children and adolescents
Regurgitation and vomiting	Hoarseness
Feeding difficulties and feeding refusal	Chronic cough
Failure to thrive	Epigastric pain and irritability
Apnea or apparent life-threatening event (ALTE)	Dysphagia
Sandifer syndrome or spasmodic torsional dystonia [8] (arching of the back and neck (Sandifer posturing) and abdominal wall contractions)	Bronchospasm and asthma

**Table 2:** Most commonly used diagnostic tests in the evaluation of GERD.

Esophageal 24 hr pH monitoring*	
Esophagram	
Upper gastrointestinal series	
Gastric emptying study	
Esophagogastroduodenoscopy (EGD)	
Esophageal impedance	
Esophageal manometry	

*Gold standard.

**Table 3:** Common indications for antireflux procedures.

Failed or refractive medical management	
Severe pulmonary disease associated with GERD*	
GERD in neurologically impaired children	
Esophageal stricture, recurrent esophagitis*, Barrett's esophagus	
Feeding disorders associated with reflux*	
Failure to thrive*	

*Most common indications for antireflux surgery in the US [10].

**Table 4:** Essential steps to the laparoscopic Nissen. See Figures 1–3 for images of these steps.

Gastroesophageal junction (GEJ) mobilization with identification of main vagi trunks	
Hiatal dissection and creation of retroesophageal window	
Division of short gastric vessels/gastrosplenic ligament	
Crural approximation	
Creation of a 360° wrap with a bougie in place	

**Table 5:** Outcomes in antireflux surgery. Ranges based on retrospective reviews by Mattioli et al. [11], Chung and Georgeson [12], Steyaert et al. [13], and Subramaniam and Dickson [14] and randomized prospective study by Kubiak et al. [15]. Those categories with only one percentage value represent the only study that individually looked at a particular outcome category for either Nissen, Toupet, or Thal.

	Dysphagia	Postoperative complications	Recurrence rates	Repeat surgical intervention
Nissen	4% to 24%	4% to 22%	3% to 46%	2% to 14%
Toupet	2%	3% to 8%	1% to 25%	~2%–11%
Thal	2% to 22%	3%	6%–20%	10%–14%

**Table 6:** Common causes of fundoplication failure described by Hunter et al. [16].

(1) Disruption of wrap	
(2) Wrap slippage	
(3) Sliding hernia with intact wrap	
(4) Overly tight or long fundoplication	
(5) Intrathoracic herniation of wrap (paraesophageal hernia)	
(6) Twisted wrap	
